# A Multi-Gene Matrix for Precision Breeding of Rice Grain Quality

**DOI:** 10.3390/plants15182753

**Published:** 2026-09-09

**Authors:** Zhixing Jin, Haipeng Tang, Xiaoyan Li, Shuxiang He, Yuhao Fu, Jun Zhu, Tao Luo

**Affiliations:** 1 Neijiang Academy of Agricultural Sciences, Neijiang 641000, China; 2 Rice Research Institute, Sichuan Agricultural University, Chengdu 611130, China

**Keywords:** rice, grain quality, multi-gene matrix, NY/T 593-2021, haplotype design, near-isogenic line, KASP

## Abstract

Rice grain quality is governed by a network of interacting loci, which makes the simultaneous improvement of several grading indices difficult. We phenotyped 240 *indica* accessions for 12 quality traits and graded six core indices—head rice rate, chalkiness degree, transparency, amylose content, gel consistency, and alkali spreading value—against the Chinese national standard NY/T 593-2021. Pairwise correlations resolved the traits into grain-shape, chalkiness, and starch clusters and quantified the trade-offs between them. KASP markers for *GS3*, *GW5*, *Chalk5*, *Wx*, and *ALK* were then used to test each locus against the six indices. Head rice rate responded to both chalkiness and grain shape; chalkiness degree was governed mainly by *Chalk5* and modified by the grain-shape loci; transparency depended on *Wx*/*ALK* and on chalkiness; amylose content and alkali spreading value tracked *Wx* and *ALK*, respectively; and gel consistency reflected the *Wx*–*ALK* combination. The haplotype *GW5*/*GS3*/*Chalk5*^L^/*Wx*^b^/*ALK*^b^ reached grade 2 or better on all six indices but occurred in only two of the 240 accessions. Near-isogenic lines carrying *GW5*/*GS3*/*Chalk5*^L^ in the Xibaizhan background produced longer, narrower grains and reduced the chalkiness degree from 17.1% to 5.3%, confirming the contribution of the appearance module within the matrix.

## 1. Introduction

Rice (*Oryza sativa* L.) feeds more than half of the world’s population, and grain quality has moved from a secondary consideration to a primary breeding objective as diets have shifted toward premium grain. The Chinese national standard for cooking rice variety quality, NY/T 593-2021, grades varieties on six indices—head rice rate, chalkiness degree, transparency, amylose content, gel consistency and alkali spreading value—which collectively cover milling, appearance, and eating quality [1,2,3,4].

These are quantitative traits, and represent trade-offs with one another. Slender grains look better but mill worse, because a high length-to-width ratio raises the risk of breakage during hulling and polishing [5,6]. Amylose content in the low-to-intermediate range improves palatability, yet grains with altered starch composition differ in fissure resistance and therefore in head rice yield [7,8]. Chalkiness is the most damaging of the three: a chalky endosperm is mechanically weak, so it lowers head rice rate at the same time as it spoils appearance and reduces translucency [9,10]. Breeders working on one index in isolation have repeatedly found that another deteriorates.

Most quality breeding has therefore been organized around a small number of major loci taken one at a time—*Wx* for amylose content, *ALK* for gelatinisation temperature, and *Chalk5* for chalkiness [11,12,13]. This approach is effective for individual traits but does not adequately address the grading standard as a whole. Introducing the rare *Wx*^mw^ allele, for instance, lowers amylose content and softens gel consistency and also improves endosperm transparency, but it leaves chalkiness unaddressed [14]; conversely, a low-chalkiness allele on its own cannot lift a line to a premium grade if its cooking quality is poor [15]. What is needed is a description of how the loci behave in combination. Based on this, Lou et al. (2026) proposed a breeding strategy that utilizes the genetic diversity of the Wx gene to analyze multi-omics modules and integrate genes related to aroma, nitrogen utilization, grain shape, chalkiness, and sugar transport, in order to balance the yield and flavour quality of rice [16].

Marker-assisted pyramiding has begun to provide insights into these interactions, but published work has generally targeted one quality dimension at a time and has not evaluated against a complete grading standard [17,18]. In particular, there has been no study that has systematically quantified the combined contribution of these five main loci (*GS3*, *GW5*, *Chalk5*, *Wx*, and *ALK*) to all six grading indicators of NY/T 593-2021 within a unified population. Here we treat these five loci as a matrix, estimate the contribution of each to each of the six NY/T 593-2021 indices in a 240-accession *indica* panel, identify the haplotype combination that satisfies all six simultaneously, and test the appearance module of that combination in near-isogenic lines.

## 2. Results

### 2.1. Single-Index Shortfalls Constrain the Overall Quality Grade

Twelve quality traits were measured on 240 *indica* accessions comprising modern cultivars and landraces (Table 1). Variation was wide overall but very unevenly distributed among traits: chalkiness degree ranged from 0.30% to 69.88% (CV = 81.43%), while brown rice rate spanned only 74.96–82.44% (CV = 1.63%). Chalky grain rate, transparency grade, and alkali spreading value were also highly variable (CV = 61.48%, 47.04% and 37.17%).

**Figure 1:**
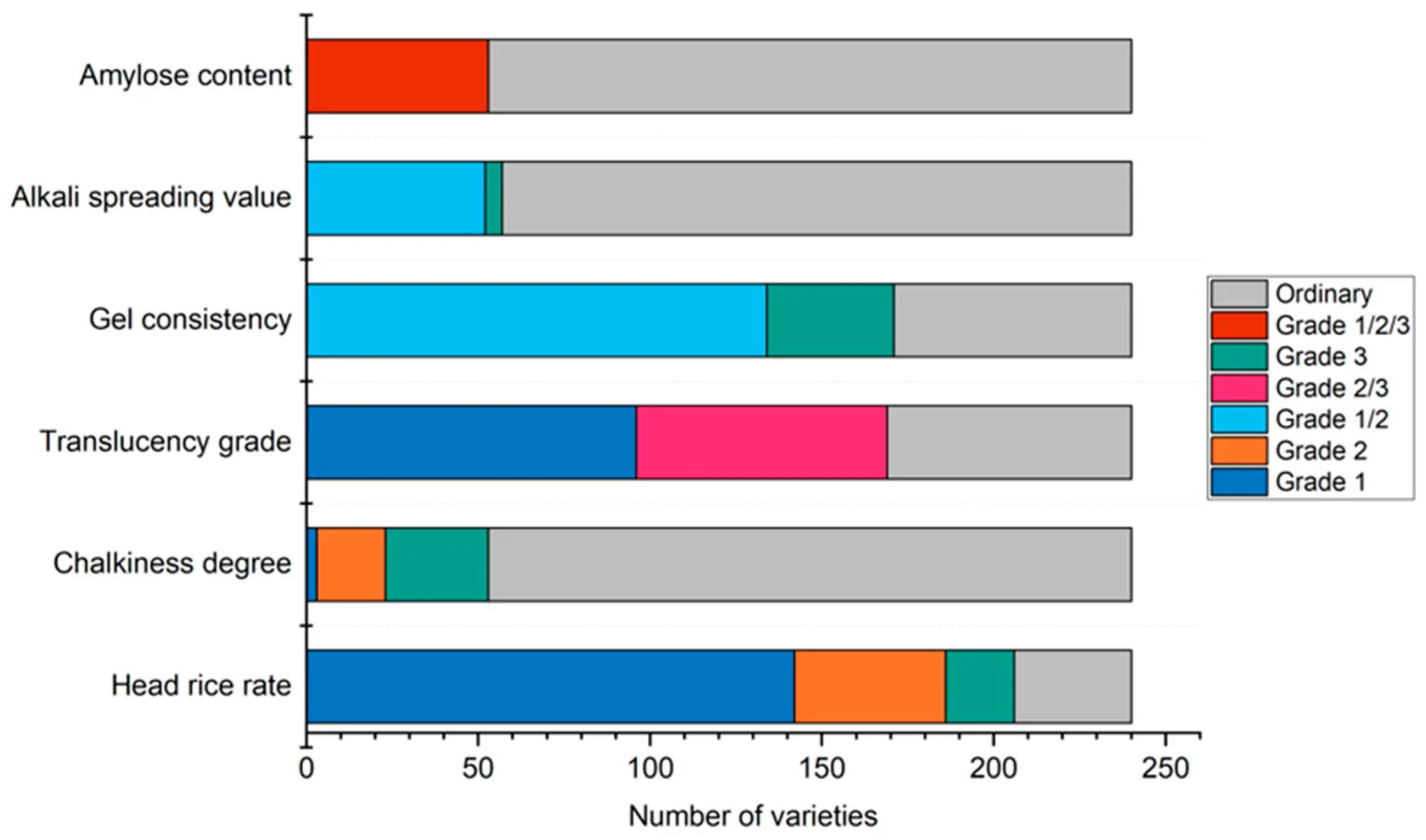
Distribution of NY/T 593-2021 quality grades across the 240-accession panel for the six core indices. Bars are stacked by grade; “Ordinary” denotes accessions falling outside the graded range for that index. Composite categories (e.g., grade 1/2) are used where the standard does not resolve adjacent grades for that index.

Each accession was then assigned a NY/T 593-2021 grade on each of the six core indices (Figure 1). Head rice rate and gel consistency were the two indices on which the panel performed well: the majority of accessions reached grade 1 or grade 2. The other four behaved differently. For amylose content, alkali spreading value, and chalkiness degree, most accessions fell outside the graded range entirely, and transparency was split roughly evenly among grade 1, grade 2–3, and ungraded material. Because the overall grade of a variety is set by its weakest index, few accessions can qualify as premium. This pattern holds even though each individual index is well represented at the top of the range somewhere in the panel.

### 2.2. Correlation Structure Among Quality Traits

**Figure 2:**
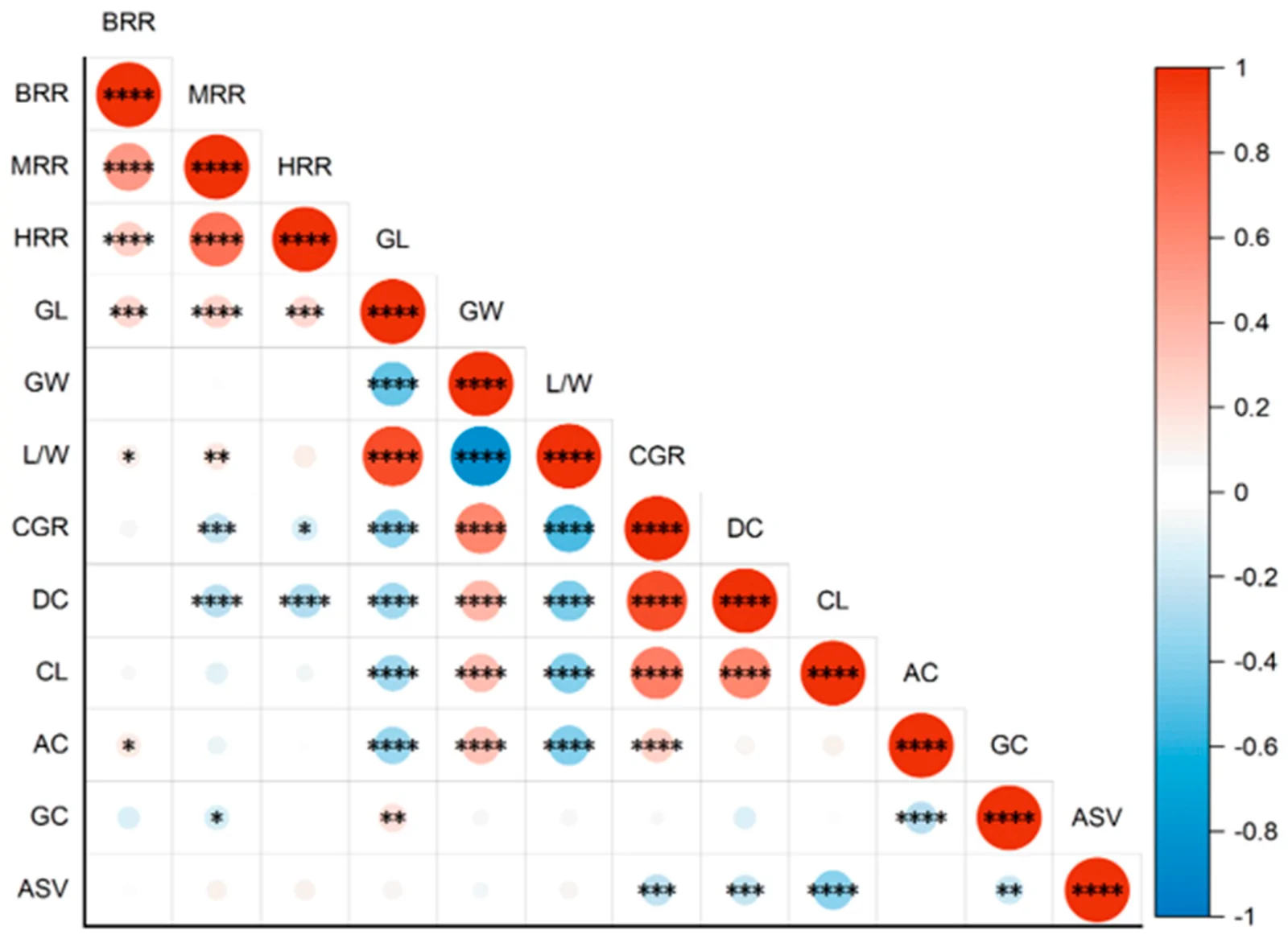
Pearson correlation matrix for 12 appearance, milling, and cooking-quality traits (*n* = 240). Circle area is proportional to |*r*|; colour indicates sign and magnitude (red, positive; blue, negative). Asterisks denote significance (* *p* ≤ 0.05, ** *p* ≤ 0.01, *** *p* ≤ 0.001, **** *p* ≤ 0.0001). BRR, brown rice rate; MRR, milled rice rate; HRR, head rice rate; GL, grain length; GW, grain width; L/W, length/width ratio; CGR, chalky grain rate; DC, chalkiness degree; CL, transparency grade; AC, amylose content; GC, gel consistency; ASV, alkali spreading value.

Pearson correlations were computed among the 12 traits (Figure 2). Head rice rate was negatively correlated with chalkiness degree (*r* = −0.27, *p* ≤ 0.0001) and positively correlated with grain length (*r* = 0.23, *p* ≤ 0.001), placing chalkiness ahead of grain shape as the dominant constraint on milling quality. Chalkiness degree fell as the length-to-width ratio rose (*r* = −0.40, *p* ≤ 0.0001), consistent with the low chalkiness of slender grains, and was also negatively correlated with alkali spreading value (*r* = −0.21, *p* ≤ 0.001), indicating a statistical association between chalkiness deposition and the fine structure of starch.

Transparency grade rose steeply with chalkiness degree (*r* = 0.62, *p* ≤ 0.0001) and fell with alkali spreading value (*r* = −0.24, *p* ≤ 0.0001); since a higher transparency score means a more opaque grain, loss of translucency accompanies both chalk accumulation and reduced starch crystallinity. Gel consistency was most closely tied to amylose content (*r* = −0.25, *p* ≤ 0.0001) and secondarily to alkali spreading value (*r* = −0.20, *p* ≤ 0.01), indicating that it is set by amylopectin branching as well as by the amylose fraction.

From a breeding perspective, grain shape is a promising target for simultaneously improving multiple traits, as it shows statistically significant correlations with chalkiness, head rice rate, and transparency in this population. Amylose content and amylopectin structure can then be tuned independently.

### 2.3. Independent Effects of the Five Major Loci

**Figure 3:**
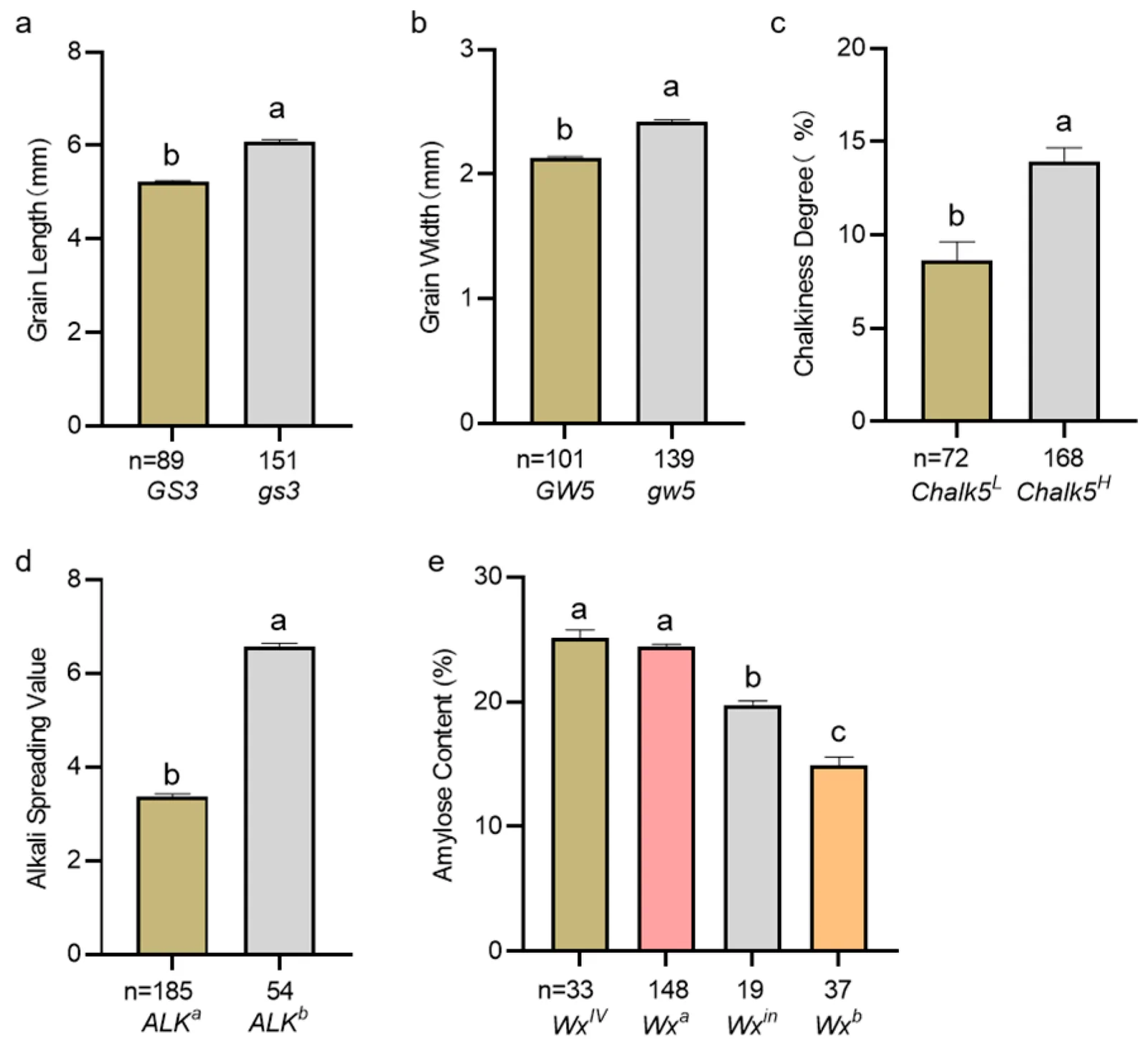
Independent effects of allelic variation at five major quality loci. Mean trait values by genotype for grain length (**a**), grain width (**b**), chalkiness degree (**c**), alkali spreading value (**d**), and amylose content (**e**). n represents the number of haplotype materials. Error bars are standard errors; different lowercase letters above bars indicate significant differences (*p* ≤ 0.05).

KASP markers were developed for the grain-length locus *GS3*, the grain-width locus *GW5*, the chalkiness locus *Chalk5*, the gelatinisation-temperature locus *ALK*, and the amylose locus *Wx* (Table S1). Each was tested against its primary trait across the panel (Figure 3). The loss-of-function alleles GS3 and GW5 increased grain length and grain width, respectively, as expected for the principal grain-shape determinants. The high-expression allele *Chalk5^H^* raised chalkiness degree from 8.6% to 13.9%. *ALK*^b^ nearly doubled the alkali spreading value relative to *ALK*^a^ (6.6 versus 3.3), indicating a lower gelatinisation temperature. The *Wx* allelic series produced a graded response in amylose content, descending from *Wx*^lv^ (25.1%) to *Wx*^a^ (24.3%) through *Wx*^in^ (19.7%) and *Wx*^b^ (14.9%).

### 2.4. GW5/GS3/Chalk5^L^/Wx^b^/ALK^b^ Is the Best-Performing Haplotype

**Figure 4:**
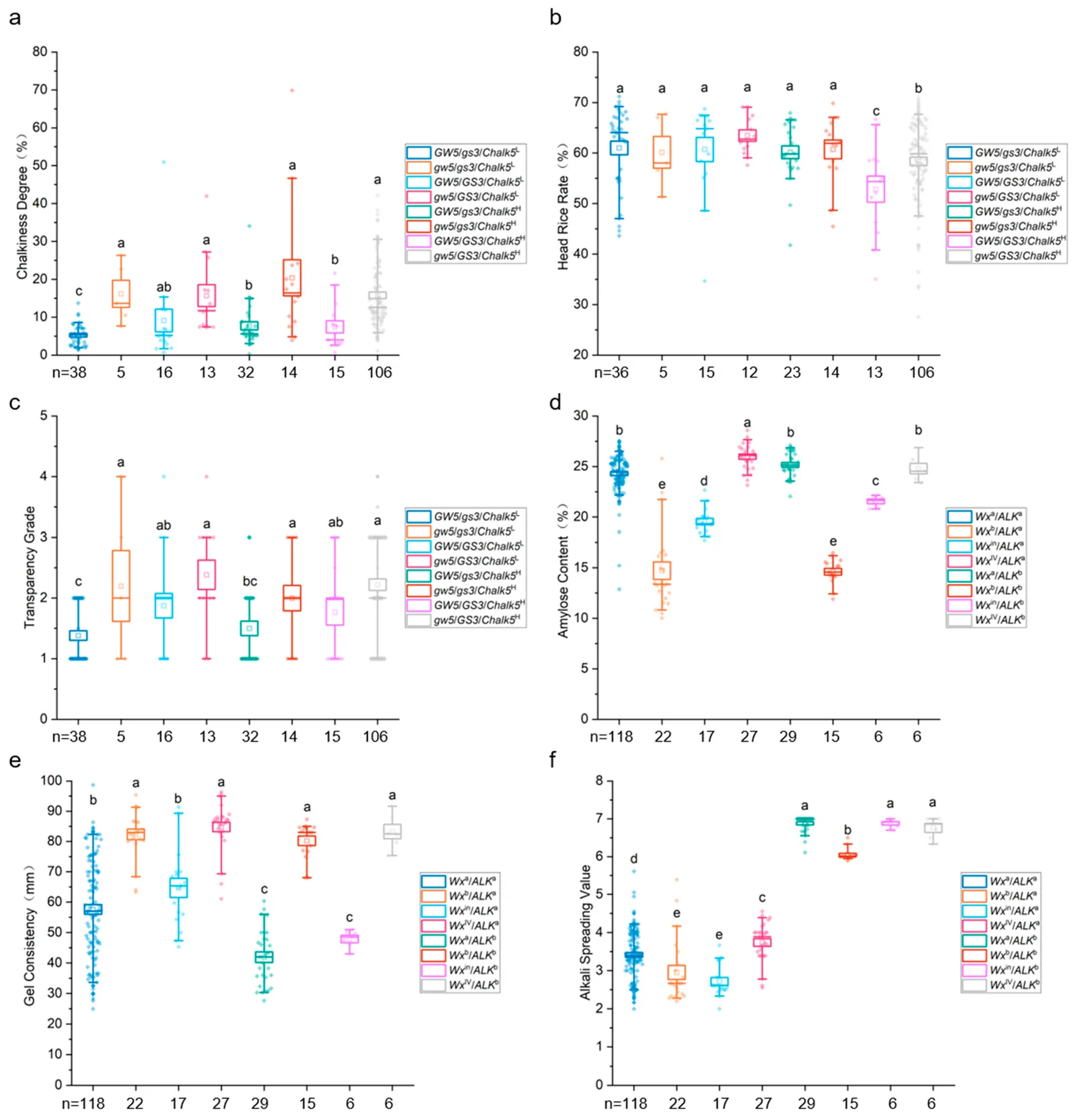
Effects of locus combinations on grain quality. *GW5*/*GS3*/*Chalk5* classes are shown for chalkiness degree (**a**), head rice rate (**b**), and transparency grade (**c**); *Wx*/*ALK* classes for amylose content (**d**), gel consistency (**e**), and alkali spreading value (**f**). Boxes show the interquartile range, whiskers the data range, and points individual accessions. n represents the number of haplotype combination materials. Different lowercase letters indicate significant differences between classes (*p* ≤ 0.05).

The three appearance and milling loci (*GS3*, *GW5*, *Chalk5*) and the two cooking-quality loci (*ALK*, *Wx*) were then analyzed as two sets of combinations (Figure 4). Among the eight three-locus classes, *GW5*/*GS3*/*Chalk5*^L^ carried the lowest chalkiness degree (median ≈ 5%) and the best transparency grade, while its head rice rate did not differ from the other classes except *GW5*/*GS3*/*Chalk5*^H^, which was the poorest. In other words, this combination lowered chalkiness and improved translucency without the loss of milling yield that the negative correlation between grain length and head rice rate would predict.

The *Wx*/*ALK* classes separated cleanly on the cooking-quality indices. *Wx*^a^/*ALK*^a^ combined high amylose content (≈24%) with firm gel consistency (≈57 mm) and a low alkali spreading value; *Wx*^b^/*ALK*^b^ gave the opposite profile (amylose ≈ 15%, gel consistency ≈ 80 mm, alkali spreading value ≈ 6). Gel consistency was not predicted by *Wx* alone: *Wx*^a^ lines were soft with *ALK*^a^ but firm with *ALK*^b^, so *ALK* acts as a modifier of gel consistency independently of its effect on gelatinisation temperature [19]. Within-class spread was small for most combinations, although *Wx*^a^/*ALK*^a^ gel consistency remained broad, indicating additional segregating variation in that class.

On this basis, the optimal haplotype was defined as *Wx*^b^ (low amylose, soft gel), *ALK*^b^ (high alkali spreading value), and *GW5*/*GS3*/*Chalk5*^L^ (low chalkiness, high head rice rate, good transparency). Only two of the 240 accessions carried the complete combination (Table 2). Their means were 67.32 ± 1.11% for head rice rate, 1.76 ± 0.62% for chalkiness degree, grade 1.00 ± 0.00 for transparency, 14.84 ± 0.83% for amylose content, 84.50 ± 0.24 mm for gel consistency, and 5.97 ± 0.04 for alkali spreading value. All six indices reached grade 2 or better under NY/T 593-2021.

### 2.5. Near-Isogenic Lines Confirm the Appearance Module

The *Wx*^b^/*ALK*^b^ combination accounts for three of the six indices directly (amylose content, gel consistency, alkali spreading value), and head rice rate and transparency both depend on chalkiness. To test the *GW5*/*GS3*/*Chalk5*^L^ contribution in a fixed background, near-isogenic lines were developed by backcrossing the low-chalkiness donor Chenghui 448 (*GW5*/*GS3*/*Chalk5*^L^) into the high-chalkiness recurrent parent Xibaizhan (*GW5*/*GS3*/*Chalk5*^H^) for three generations.

**Figure 5:**
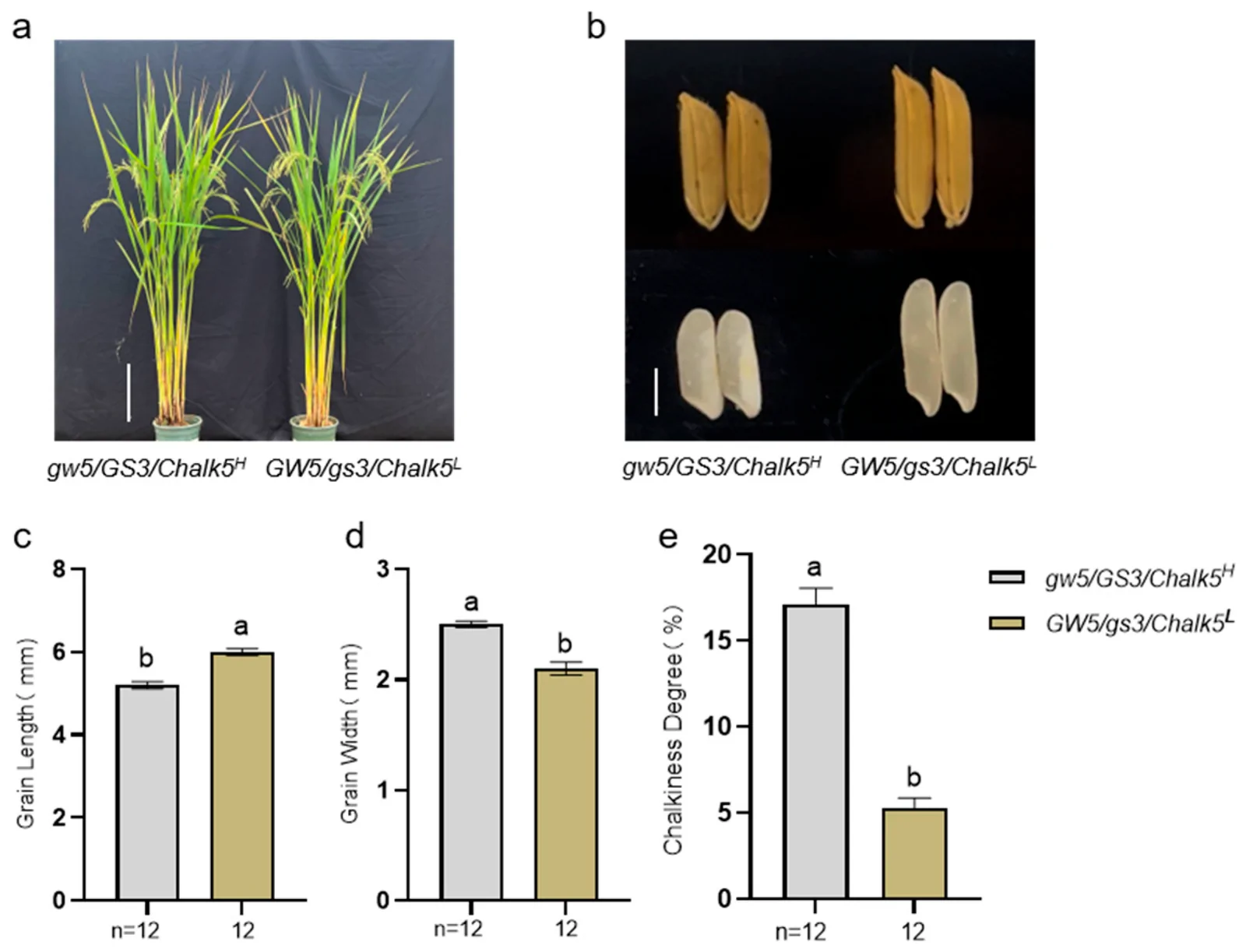
Comparative phenotypes of the near-isogenic lines (NILs). (**a**) Plant architecture at physiological maturity; scale bar, 20 cm. (**b**) Grain morphology; upper row, brown rice; lower row, milled rice; scale bar, 2 mm. (**c**–**e**) Grain length, grain width, and chalkiness degree. n represents the number of materials for the genetic module detection. Error bars are standard errors; different lowercase letters above bars indicate significant differences (*p* ≤ 0.05).

The two lines were indistinguishable in plant architecture and other agronomic traits (Figure 5a) but differed in grain appearance. The *GW5*/*GS3*/*Chalk5*^L^ line produced slender grains 6.0 mm long and 2.1 mm wide, against 5.2 mm and 2.5 mm for *GW5*/*GS3*/*Chalk5*^H^ (Figure 5b–d). Chalkiness degree fell from 17.1% to 5.3%, a reduction of roughly two thirds (Figure 5e).

## 3. Discussion

Treating five major loci as a matrix rather than as separate targets identified one combination, *GW5*/*GS3*/*Chalk5*^L^/*Wx*^b^/*ALK*^b^, that satisfies all six NY/T 593-2021 indices at grade 2 or better. That the combination occurred in only two of 240 accessions limits the statistical power for promoting this discovery. However, the rarity of this haplotype itself highlights the value of marker-assisted polymorphism. Before reaching a definite conclusion about its wide applicability, we need to conduct further verification in larger and more diverse populations.

Several limitations qualify these results. The near-isogenic lines test only the appearance and milling module of the haplotype; the recurrent parent supplied the *Wx* and *ALK* alleles, so the contribution of *Wx*^b^/*ALK*^b^ in that background has not been validated directly, and the NILs were scored for grain dimensions and chalkiness rather than for all six indices. Confirming the full combination will require introgressing all five loci into a common background and grading the resulting lines against the standard.

The five loci also do not account for the whole correlation structure. Neither the chalkiness–alkali spreading value association (*r* = −0.21) nor the transparency–chalkiness association (*r* = 0.62) is fully explained by the *Chalk5*, *Wx*, and *ALK* genotype. Chalk deposition and the thermal properties of starch may share upstream control through carbon–nitrogen partitioning during grain filling [20], and loss of translucency plausibly arises by two routes—optical scattering from chalky tissue and defects in starch packing—that respond to different genes [21]. If so, transparency will not be corrected by manipulating a single pathway. Studies have shown that genes such as *GL7* and *GW8* related to appearance quality can significantly improve grain shape and reduce the mottling degree of rice. However, their distribution frequency in this study was too low to allow for statistical analysis. In the future, they can be incorporated as potential genes into the quality gene module, which is expected to further enhance the quality of rice [22].

Whether the optimal haplotype carries a yield penalty is untested, as is its stability across environments; the panel was grown at a single site in one season, and both chalkiness and gelatinisation temperature are strongly temperature-sensitive. Multi-site, multi-season trials are needed before the combination can be recommended for deployment. Beyond the five major loci, minor-effect modifiers and genotype-by-environment interactions were not modelled, which limits how far the haplotype can be expected to transfer to other genetic backgrounds. The obvious next step is to use the existing NILs for transcriptome and metabolome profiling during grain filling, and to combine this with association mapping of minor-effect loci, so that the current major-gene matrix can be extended into a model that includes modifier networks and environmental response.

## 4. Materials and Methods

### 4.1. Plant Materials

The 240 *indica* accessions used in this study were provided by Sichuan Agricultural University and grown in 2023 at the rice breeding station in Neijiang, Sichuan, China. A total of 36 plants of each material were planted. The row and plant spacing was 30 cm × 20 cm. A randomized complete block design was adopted for planting. Each material was replicated three times. Standard agronomic management measures (including fertilization, irrigation, and pest control) were implemented.

### 4.2. KASP Genotyping

KASP (Kompetitive Allele-Specific PCR) genotyping resolves single-nucleotide polymorphisms and small insertions/deletions using two allele-specific forward primers carrying distinct tail sequences, a common reverse primer, a FRET cassette, and Taq DNA polymerase. Allelic variant information for *GS3*, *GW5*, *Chalk5*, *Wx*, and *ALK* was retrieved from RiceNavi; polymorphic sites were confirmed by BLAST and sequence alignment, and primers were designed in Primer3Plus (https://primer3plus.com, accessed on 1 March 2023). Marker sequences and the corresponding variant sites are given in Table S1. Amplification and allele calling followed established KASP protocols [23].

### 4.3. Development of Near-Isogenic Lines

Near-isogenic lines were developed by marker-assisted backcrossing, with Xibaizhan as the recurrent parent and Chenghui 448 as the donor of *GW5*, *GS3*, and *Chalk5*^L^. The F_1_ was backcrossed to Xibaizhan for three generations, with foreground selection using the functional markers in each backcross generation, and individual plants with similar agronomic traits to the maternal parent Xibaizhan as the backcrossing targets. Heterozygous BC_3_F_1_ plants were self-pollinated; homozygous target genotypes were selected from the resulting BC_3_F_2_ population and advanced by selfing to BC_3_F_4_, yielding lines that differ from the recurrent parent at the *GW5*, *GS3*, and *Chalk5* loci.

### 4.4. Phenotyping

Each accession was harvested separately, air-dried in mesh bags to below 14.5% moisture, and stored at room temperature for three months so that physicochemical properties stabilized. The rice was finely ground using the 6N80 rice mill, separated by the FQS-13 × 20 rice separation device, and processed to meet the national standard precision requirements for rice. One hundred head rice grains per accession were scanned on an SC-G automatic seed analyzer and thousand-grain-weight analyzer (Wanshen Detection Technology, Hangzhou, China) to obtain grain length and width; the length-to-width ratio was calculated from the means of the 100 grains. Milling-quality indices were determined according to NY/T 593-2021 [4]. Chalky grain rate and chalkiness degree were measured on 100 fully filled milled grains using a MICROTEK scanner (MRS 9600TFU2L) with a Wanshen SC-E rice appearance analyzer. Amylose content, gel consistency, and gelatinisation temperature (scored as alkali spreading value) were determined as previously described [19].

### 4.5. Statistical Analysis

Genotype and phenotype data for the 240 accessions were compiled in Microsoft Excel. Differences among genotype classes were tested by one-way analysis of variance followed by Duncan’s multiple range test at *p* ≤ 0.05; pairwise contrasts were tested by Student’s *t*-test. Pearson correlation coefficients were computed among the 12 quality traits. Figures were prepared in Origin 2024.

## 5. Conclusions

Correlation analysis of 12 quality traits in 240 *indica* accessions, combined with KASP genotyping at five major quality loci, resolved how each locus contributes to the six indices of NY/T 593-2021 and identified *GW5*/*GS3*/*Chalk5*^L^/*Wx*^b^/*ALK*^b^ as the haplotype that satisfies all six simultaneously. Near-isogenic lines confirmed that the *GW5*/*GS3*/*Chalk5*^L^ module alone reduces chalkiness degree substantially in a fixed background. The five markers reported here can be applied directly in marker-assisted selection for grain quality in *indica* breeding programmes.

## Figures and Tables

**Table 1:** Descriptive statistics for 12 grain quality traits in the 240-accession *indica* panel.

Trait	Max.	Min.	Mean	SD	CV (%)
Brown rice rate (%)	82.44 ± 0.29	74.96 ± 1.56	79.72 ± 0.59	1.30 ± 0.10	1.63 ± 0.04
Milled rice rate (%)	74.41 ± 3.69	46.06 ± 1.65	67.57 ± 3.08	3.92 ± 0.73	5.80 ± 0.22
Head rice rate (%)	71.19 ± 3.39	27.60 ± 3.26	59.27 ± 4.91	8.19 ± 1.02	13.82 ± 1.29
Grain length (mm)	6.89 ± 0.57	4.42 ± 0.97	5.55 ± 0.24	0.53 ± 0.05	9.57 ± 1.77
Grain width (mm)	2.82 ± 0.29	1.74 ± 0.30	2.30 ± 0.12	0.20 ± 0.01	8.65 ± 0.80
Length/width ratio	3.41 ± 0.62	1.75 ± 0.35	2.45 ± 0.16	0.39 ± 0.00	15.88 ± 4.63
Chalky grain rate (%)	98.65 ± 1.00	1.07 ± 0.21	41.99 ± 4.87	25.82 ± 2.32	61.48 ± 9.53
Chalkiness degree (%)	69.88 ± 6.83	0.30 ± 0.14	12.30 ± 3.03	10.02 ± 1.46	81.43 ± 4.69
Transparency grade	4.00 ± 0.13	1.00 ± 0.00	1.94 ± 0.15	0.91 ± 0.03	47.04 ± 1.11
Amylose content (%)	28.59 ± 1.34	7.87 ± 0.93	22.63 ± 1.28	4.44 ± 0.29	19.61 ± 1.04
Gel consistency (mm)	98.67 ± 1.04	25.00 ± 3.00	63.44 ± 4.71	19.35 ± 1.45	30.50 ± 4.27
Alkali spreading value	7.00 ± 0.65	2.00 ± 0.31	4.09 ± 0.35	1.52 ± 0.17	37.17 ± 2.90

SD, standard deviation; CV, coefficient of variation. Transparency is scored on the NY/T 593-2021 1–4 scale, on which a higher score denotes a less translucent grain. Data are means ± standard deviations, *n* = 12.

**Table 2:** Quality performance of the two accessions carrying the complete optimal haplotype.

Accession	Genotype	Head Rice Rate (%)	Chalkiness Degree (%)	Transparency Grade	Amylose Content (%)	Gel Consistency (mm)	Alkali Spreading Value	Grade
4484	*GW5*/*GS3*/*Chalk5*^L^/*Wx*^b^/*ALK*^b^	68.10 ± 2.18	2.21 ± 0.31	1.00 ± 0.00	15.43 ± 1.16	84.67 ± 1.48	5.94 ± 0.03	2
Chenghui 448	*GW5*/*GS3*/*Chalk5*^L^/*Wx*^b^/*ALK*^b^	66.53 ± 2.52	1.34 ± 0.10	1.00 ± 0.00	14.25 ± 2.17	84.33 ± 1.57	6.00 ± 0.03	2

Grade is the overall NY/T 593-2021 grade, set by the weakest of the six indices. Data are means ± standard deviations, *n* = 12.

## Data Availability

The data presented in this study are available upon request from the corresponding authors. The data are not publicly available due to privacy and ethical restrictions.

## Supplementary Materials

The following supporting information can be downloaded at: https://www.mdpi.com/article/10.3390/plants15182753/s1, Table S1: KASP marker information for the five target loci.
